# Ventricular Strain Predicts the Risk of Right Heart Failure After Durable Left Ventricular Assist Device Implantation

**DOI:** 10.1016/j.jacadv.2026.103240

**Published:** 2026-09-17

**Authors:** Michal Schäfer, Syed Shahyan Bakhtiyar, Thomas Hanff, Spencer Carter, Satvik Ramakrishna, Ethan Tumarkin, Matthew L. Goodwin, Hiroshi Kagawa, Bryce Hill, Andrew Scott, Stavros Drakos, Josef Stehlik, Craig H. Selzman

**Affiliations:** a Division of Cardiothoracic Surgery, University of Utah, Salt Lake City, Utah, USA; b Division of Cardiology, University of Utah, Salt Lake City, Utah, USA; c Division of Cardiology, George E. Wahlen Department of Veterans Affairs Medical Center, Salt Lake City, Utah, USA; d Department of Anesthesiology, University of Utah, Salt Lake City, Utah, USA

**Keywords:** LVAD, right heart failure, RVPA coupling, strain

## Abstract

**Background:**

Right heart failure (RHF) is prevalent after left ventricular assist device (LVAD) implantation and associated with poor short- and long-term outcomes. The interaction of the right ventricle (RV) with its afterload before LVAD implantation may impact the incidence of RHF.

**Objectives:**

The aim of the study was to investigate RV pulmonary artery (RVPA) coupling indexes derived from fusing myocardial strain imaging and invasive hemodynamics for their potential to predict RHF or the need for an RV assist device (RVAD).

**Methods:**

Patients (N = 288) underwent a retrospective imaging analysis to yield RVPA coupling indexes fused from right ventricle free-wall (RVFW) strain or right ventricle global longitudinal strain (RVGLS) with systolic pulmonary artery pressure (sPAP). Multivariable logistic regression models considering the RVPA indexes and standard hemodynamics were assessed to predict: 1) the need for an RVAD; and 2) RHF.

**Results:**

Eighty-two patients (28.5%) developed RHF, and 52 (18.1%) required a temporary RVAD. Reduced RVFW strain (OR: 1.37; 95% CI: 1.23-1.55; *P* < 0.001) and RVGLS strain (OR: 1.55; 95% CI: 1.34-1.83; *P* < 0.001) were associated with the need for an RVAD. RVPA coupling defined by RVFW/sPAP (OR: 0.03^−4^; 95% CI: 0.03^−6^ to 0.01^−2^; *P* < 0.001) and by RVGLS/sPAP (OR: 0.09^−6^; 95% CI: 0.01^−9^ to 0.06^−4^; *P* < 0.001) were both associated with the need for an RVAD. The multivariable model with the highest predictive accuracy consisted of RVFW strain and pulmonary artery pulsatility index, yielding an area under the curve of 0.850 with a sensitivity of 88.6% and a specificity of 71.3%.

**Conclusions:**

RVFW strain and coupling indexes derived from catheterization and echocardiographic RV strain imaging can aid with the need for an RVAD and RHF risk stratification following LVAD implantation.

Right heart failure (RHF) is a critical complication following durable mechanical circulatory support (MCS) device implantation.^1^ Contemporary studies report an incidence between 15% and 45%, with the variation mostly dependent on the definition of RHF.^2^, ^3^, ^4^ Depending on the etiology of RHF, therapeutic options include increasing mean arterial pressure to improve right ventricle (RV) perfusion, adding inhaled nitric oxide to reduce RV afterload, adjusting ventilator settings, using inotropic agents, and using a right ventricular assist device (RVAD). Furthermore, acute RHF has been shown to have critical prognostic value for predicting long-term clinical outcomes.^3^ As a result, multiple risk scores and calculators have been developed to estimate the preoperative risk of RHF. Unfortunately, none of the proposed risk models include a quantitative imaging assessment of RV function.

RV myocardial strain and right ventricle pulmonary arterial coupling (RVPA) have been extensively studied as a hemodynamic predictor of RHF in patients with primary pulmonary hypertension, but their application in studying RV response to loading conditions before and after MCS implementations has been limited.^5^^,^^6^ Regarding RVPA coupling, the traditional and most accurate measurement of the ratio of the RV contractility to its afterload is derived from a pressure-volume loop analysis and has been explored only in MCS studies post implantation in a smaller series.^7^^,^^8^ However, this method is not feasible for routine clinical care because of the specialized equipment and labor required. Therefore, multiple noninvasive imaging-based indexes have been proposed to aid in the measurement of RV-PA coupling.^9^^,^^10^ Most recently, RV strain-based measurements serving as the input for contractility in the RVPA coupling ratio have shown promising results toward predicting clinical outcomes.^11^ Systolic pulmonary arterial pressure (sPAP) is typically estimated from transthoracic echocardiography (TTE) and serves as an input for the afterload in the RVPA coupling ratio.^11^^,^^12^ However, a combination of RV strain-based deformation and direct pressure measurements from catheterization to yield hybrid imaging-invasive RVPA coupling indexes has not yet been explored.

Consequently, in this study, we sought to combine echocardiography-based strain measurements, both individually and in combination, with right heart catheterization (RHC) hemodynamic indexes to predict the need for an RVAD or RHF immediately after durable MCS implantation.

## Methods

This was a single-institution, retrospective cohort study of all patients who underwent left ventricular assist device (LVAD) implantation between January 2015 and March 2024, and who were managed by the Utah Transplantation Affiliated Hospitals Cardiac Transplant Program (University of Utah and the George E. Wahlen Department of the Veterans Affairs Medical Center). TTE and RHC indexes were obtained as part of the standardized pre-MCS evaluation and were typically performed within 48 hours of each other, and within 1 week before implantation. The primary outcomes were: 1) an unplanned need for an RVAD (surgically or percutaneously implanted MCS within 30 days postoperatively); and 2) RHF, defined as the composite outcome of any of the following: requirement for inotropic therapy for >14 days, use of inhaled nitric oxide for >48 hours, or need for an RVAD.^2^ Both primary outcomes represent categorical conditionals describing the early acute RHF or early postimplant RHF as described by the MCS academic research consortium and as recently applied in the STOP-right ventricular failure risk calculator development.^2^^,^^13^ The secondary outcome was all-cause mortality. This study was approved by the local institutional review board, and written consent was obtained from all patients where applicable. The study complied with the Declaration of Helsinki.

### RVPA coupling indexes

Noninvasive RVPA coupling indexes derived from echocardiographic measurements are typically derived from the trans-annular plane excursion (TAPSE) or right ventricular free-wall (RVFW) strain, with the sPAP estimate serving as the input for the RV afterload.^11^^,^^12^ sPAP is typically estimated from the tricuspid regurgitation gradient and right atrial pressure. In this study, we explored RVPA coupling derived from preoperative TTE. Using the TTE, RVFW strain was measured in a standard fashion from the RV-focused apical 4-chamber view using the 3-segment approach, which measures strain in the basal, midventricular, and apical regions and averages the free wall value.^14^ In addition to the RVFW strain, we also sampled the RV global longitudinal strain (RVGLS). sPAP derived from the preoperative RHC was then combined with strain indexes to yield the final RVPA coupling indexes: 1) RVFW/sPAP; and 2) RVGLS/sPAP. We also analyzed TTE-based RVPA coupling using the TAPSE/sPAP ratio.

All strain measurements were analyzed retrospectively by exporting archived images to a commercially available software program for speckle-tracking analysis (TomTec Cardiac Performance Analysis). All strain parameters were collected using the semiautomatic contour-detection mode for endocardial border detection, with the tracking revision checked in each subject and revised as needed. Strain parameters were collected over 3 cardiac cycles, and the average value for each variable was reported.

### Statistical analysis

All variables were checked for the distributional normality assumption using normal plots and Kolmogorov-Smirnov and Shapiro-Wilks tests. Comparative analyses were performed using a 2-sample independent *t*-test for continuous, normally distributed variables or the Wilcoxon rank sum test for non-normally distributed variables. The chi-square test was used for categorical data. To test the association between RV imaging and RHC biomarkers and post-MCS-implantation RHF or the need for an RVAD, all aforementioned indexes were subjected to a simple univariable logistic regression analysis. All variables significantly associated with the need for an RVAD and/or RHF were included in a multivariable logistic regression model using a backward elimination stepwise approach. Catheterization and imaging-based variables were initially considered for multivariable logistic regression and were all from univariable logistic regression with *P* values <0.05. Variables included in multivariable models were checked for collinearity using variance inflation factors (values in the range of 1-3 were considered) and a correlation matrix. For collinear variables, the variable with the strongest correlation with the need for an RVAD and/or RHF was used in multivariable modeling. Hosmer-Lemeshow testing was done to assess the model calibration. With 52 outcome events and 3 or 4 candidate variables, the events-per-variable ratio was approximately 13, suggesting a low risk of model overfitting. Given the limited number of predictors and adequate events-per-variable ratio, backward elimination was selected to obtain a parsimonious explanatory model. Receiver operating characteristic analysis was used to assess the prognostic performance of models in both the derivation and validation cohorts. The survival analysis was conducted using the Kaplan-Meier method, and the Cox proportional hazards survival model was used for the univariable analysis of survival prognosis with censoring at 3 years. Statistical analyses were performed using Prism (version 9.4.1 or higher, GraphPad Software) and SAS (version 9.4 or higher, SAS Institute). Unless specified otherwise, all statistical tests were considered significant with a 2-sided *P* value <0.05.

## Results

**Figure 1:**
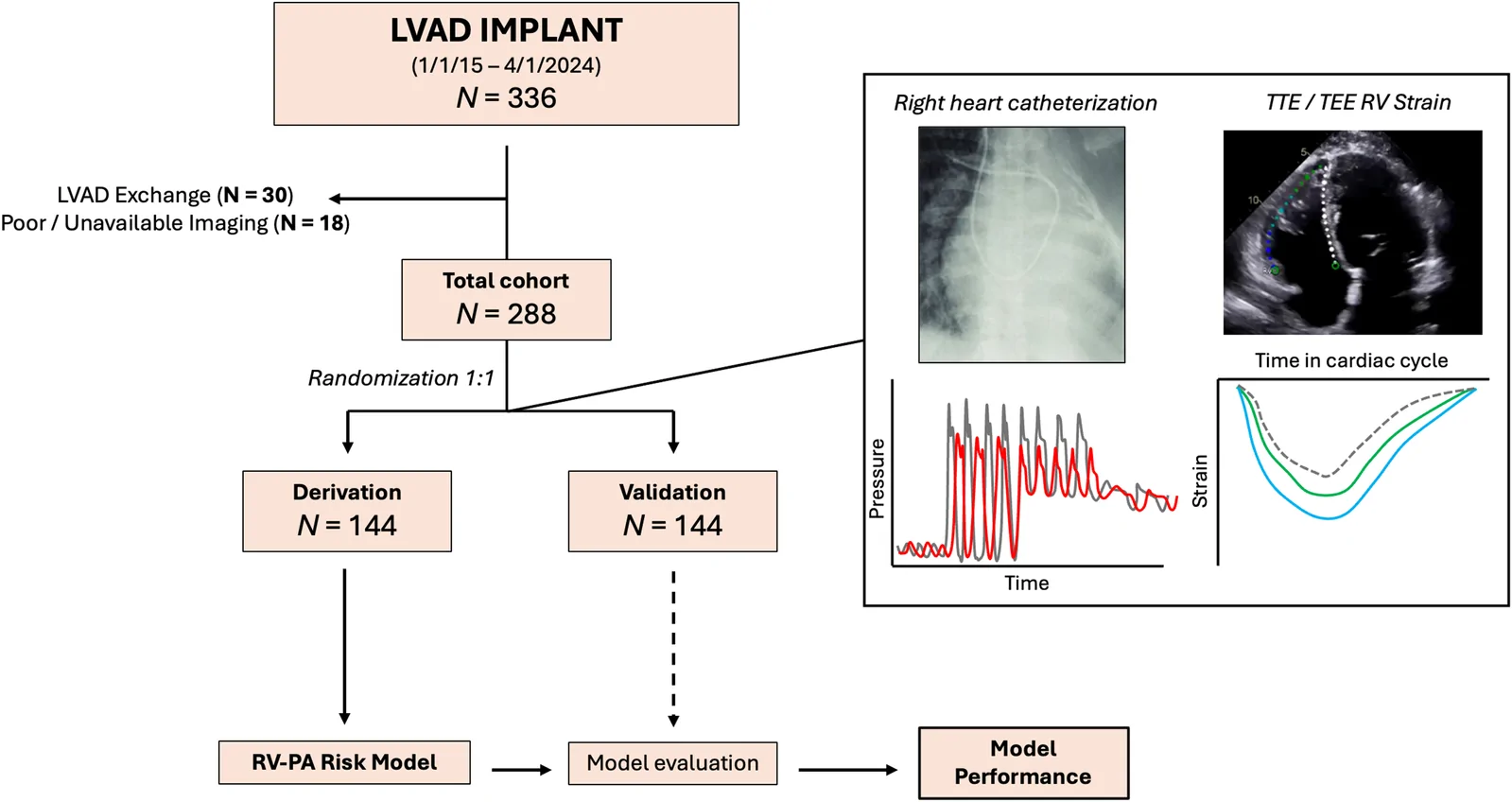
Study Flow Diagram Flow diagram depicting the overall study design and patient outcomes. We included all patients who underwent LVAD implantation from 2015 to 2024, and after the application of the exclusion criteria, we analyzed 288 patients. The set of variables used for multivariable analysis included only echocardiographic strain parameters and right heart catheterization hemodynamic indexes. LVAD = left ventricular assist device; RV-PA = right-ventricular pulmonary artery; TEE = trans-esophageal echocardiography; TTE = transthoracic echocardiography.

In total, 336 patients underwent LVAD implantation between January 2015 and April 2024. After application of the exclusion criteria (LVAD exchange, n = 30; poor imaging quality, n = 18), 288 patients were included in the study (Figure 1). Of these, 82 developed RHF (28.5%), and 52 patients (18.1%) required a temporary RVAD. The summary of patient characteristics and hemodynamics for the entire group, as well as comparisons for patients with and without RHF and requiring an RVAD, is depicted in Table 1. Considering the entire population, the mean age was 56.4 years, and 47 patients (16.3%) were female. One hundred sixty-five patients (57.3%) had nonischemic cardiomyopathy. Treatment intents at the time of implantation were destination therapy (n = 107, 37.2%), bridge-to-decision (n = 132, 45.8%), and bridge-to-transplant (n = 49, 17.0%). The median time between RHC and TTE assessment was 2 days, with an interquartile range of 0 to 7 days. Patients who required an RVAD had a higher diastolic pulmonary artery pressure (25 ± 9 mm Hg vs 29 ± 8 mm Hg, *P* = 0.002), pulmonary capillary wedge pressure (23 ± 9 mm Hg vs 25 ± 7 mm Hg, *P* = 0.029), RV end-diastolic pressure (median: 7 mm Hg vs 10 mm Hg, *P* = 0.004), right atrial pressure (median: 10 vs 14, *P* < 0.001), and lower pulmonary artery pressure index (median: 2.5 vs 1.4, *P* < 0.001). Similarly, patients who developed RHF had a higher diastolic pulmonary artery pressure (25 ± 9 mm Hg vs 28 ± 8 mm Hg, *P* = 0.004), pulmonary capillary wedge pressure (23 ± 9 mm Hg vs 25 ± 7 mm Hg, *P* = 0.020), RV end-diastolic pressure (median: 7 vs 9 mm Hg, *P* = 0.004), right atrial pressure (median: 10 vs 14, *P* < 0.001), and lower pulmonary artery pressure index (median: 2.5 vs 1.5, *P* < 0.001). Of the total, 46.2% received HeartMate 3, and there was no association between the incidence of RHF or the need for an RVAD and the type of device implanted.

**Table 1:** Patient Characteristics, Hemodynamics

	All Patients (N = 288)	Need for RVAD			Right Heart Failure		
		RVAD (−) (n = 236)	RVAD (+) (n = 52)	*P* Value	RHF (−) (n = 206)	RHF (+) (n = 82)	*P* Value
Age (y)	56.4 ± 14.2	56.5 c 14.1	55.9 ± 14.8	0.859	56.7 ± 14.5	55.6 ± 13.6	0.676
Sex (female %)	47 (16.3%)	37 (15.7%)	10 (19.2%)	0.530	35 (17.0%)	12 (14.6%)	0.625
BSA (m^2^)	2.05 ± 0.26	2.05 ± 0.25	2.06 ± 0.29	0.821	2.06 ± 0.25	2.04 ± 0.28	0.783
NICM	165 (57.3%)	132 (55.9%)	33 (63.4%)	0.302	114 (55.3%)	51 (62.2%)	0.288
Prior MCS							
IABP	16 (5.6%)	12 (5.8%)	4 (7.7%)	0.421	10 (4.8%)	15 (18.3%)	0.027
Impella	25 (8.7%)	10 (4.2%)	15 (28.8%)	0.068	10 (4.8%)	6 (7.3%)	0.410
Treatment intention							
DT	107 (37.2%)	91 (38.6%)	16 (30.8%)		79 (38.3%)	28 (34.1%)	
BTT	132 (45.8%)	106 (44.9%)	26 (50.0%)	0.571	92 (44.7%)	40 (48.8%)	0.677
BTD	49 (17.0%)	39 (16.5%)	10 (19.2%)		35 (17.0%)	14 (17.1%)	
Device							
HeartMate II	28 (9.7%)	26 (11.0%)	2 (3.8%)	0.114	23 (11.1%)	5 (6.1%)	0.190
HeartWare	116 (40.3%)	96 (40.7%)	20 (38.5%)	0.768	83 (40.3%)	33 (40.2%)	0.994
Jarvik 2000	11 (3.8%)	11 (4.7%)	0	0.372	10 (4.8%)	1 (1.2%)	0.146
Heartmate III	133 (46.2%)	103 (43.6%)	30 (57.6%)	0.310	90 (43.7%)	43 (52.4%)	0.179
RHC							
sPAP (mm Hg)	50 ± 15	50 ± 15	51 ± 15	0.666	50 ± 15	50 ± 13	0.976
dPAP (mm Hg)	26 ± 9	25 ± 9	29 ± 8	0.002	25 ± 9	28 ± 8	0.023
mPAP (mm Hg)	35 ± 10	35 ± 10	36 ± 12	0.338	35 ± 10	36 ± 11	0.386
PCWP (mm Hg)	23 ± 8	23 ± 9	25 ± 7	0.029	23 ± 9	25 ± 7	0.020
RVESP (mm Hg)	49 ± 15	49 ± 15	52 ± 13	0.176	49 ± 16	50 ± 12	0.421
RVEDP (mm Hg)	7 (4-11)	7 (4-10)	10 (6-13)	0.004	7 (3-10)	9 (6-13)	0.004
RAP (mm Hg)	11 (7-17)	10 (6-16)	14 (10-19)	<0.001	10 (6-15)	14 (10-19)	<0.001
CI (L/min/m^2^)	1.9 (1.6-2.2)	1.9 (1.6-2.2)	1.9 (1.4-2.3)	0.896	1.9 (1.6-2.2)	1.9 (1.4-2.3)	0.648
PVR (WU)	3.3 (2.2-5.0)	3.3 (2.2-4.9)	3.8 (2.0-5.3)	0.583	3.3 (2.2-4.8)	3.4 (2.1-5.2)	0.448
PAPi	2.2 (1.3-3.7)	2.5 (1.4-3.9)	1.4 (0.9-2.3)	<0.001	2.5 (1.5-4.0)	1.5 (1.0-2.6)	<0.001

Values are mean ± SD or median (IQR).

BSA = body surface area; BTD = bridge-to-decision; BTT = bridge-to-transplant; CI = cardiac index; dPAP = diastolic pulmonary pressure; DT = destination therapy; IABP = intra-aortic balloon pump; MCS = mechanical circulatory support; mPAP = mean pulmonary arterial pressure; NICM = non-ischemic cardiomyopathy; PAPi = pulmonary artery pulsatility index; PCWP = pulmonary capillary wedge pressure; PVR = pulmonary vascular resistance; RAP = right atrial pressure; RHC = right heart catheterization; RHF = right heart failure; RVAD = right ventricular assist device; RVEDP = right ventricular end-diastolic pressure; RVESP = right ventricular end-systolic pressure; sPAP = systolic pulmonary arterial pressure.

### RV strain and RVPA coupling parameters

The summary of RVPA coupling and RV strain indexes for all patients, along with comparative analyses for RHF and those requiring RVAD, is depicted in Table 2. Patients who required an RVAD had lower RVFW strain (−13.2% ± 4.8% vs −8.1% ± 2.9%, *P* < 0.001) and RVGLS (−9.8% ± 3.4% vs −6.2% ± 2.4%, *P* < 0.001). RVPA coupling derived from TTE showed decreased RVFW/sPAP (median: 0.25% vs 0.17%/mm Hg, *P* < 0.001) and RVGLS/sPAP (median: 0.18% vs 0.14%/mm Hg, *P* < 0.001) in patients who required an RVAD. Patients who developed RHF had also decreased RV strain values, including RVFW strain (−13.4% ± 4.9% vs −9.4% ± 3.6%, *P* < 0.001) and RVGLS (−8.8% ± 3.6% vs −7.2% ± 3.5%, *P* < 0.001). RVPA coupling indexes measured by TTE were also worse in patients with the RHF with decreased RVFW/sPAP (median: 0.25% vs 0.19%/mm Hg, *P* < 0.001) and RVGLS/sPAP (median: 0.18% vs 0.14%/mm Hg, *P* < 0.001). The interreader and intrareader variability, analyzed across 40 patients, yielded intraclass correlation coefficients of 0.89 and 0.93, respectively.

**Table 2:** RV-PA Coupling and RV Strain Indexes

	All Patients (N = 288)	Need for RVAD			Right Heart Failure		
		RVAD (−) (n = 236)	RVAD (+) (n = 52)	*P* Value	RHF (−) (n = 206)	RHF (+) (n = 82)	*P* Value
TAPSE (mm)	16 ± 5	16 ± 5	15 ± 5	0.256	16 ± 5	15 ± 4	0.209
sPAP (mm Hg)	44 ± 14	44 ± 15	43 ± 14	0.701	44 ± 15	44 ± 14	0.934
TAPSE/sPAP	0.35 (0.26-0.50)	0.36 (0.26-0.54)	0.33 (0.25-0.43)	0.384	0.37 (0.26-0.54)	0.33 (0.26-0.43)	0.383
Strain and RV-PA coupling							
RVFW (%)	−12.2 ± 4.9	−13.2 ± 4.8	−8.1 ± 2.9	<0.001	−13.4 ± 4.9	−9.4 ± 3.6	<0.001
RVGLS (%)	−9.1 ± 3.5	−9.8 ± 3.4	−6.2 ± 2.4	<0.001	−10.0 ± 3.5	−7.1 ± 2.7	<0.001
RVFW/sPAP (%/mm Hg)	0.23 (0.17-0.31)	0.25 (0.19-0.34)	0.17 (0.11-0.21)	<0.001	0.25 (0.19-0.36)	0.19 (0.14-0.25)	<0.001
RVGLS/sPAP (%/mm Hg)	0.17 (0.13-0.23)	0.18 (0.14-0.25)	0.13 (0.09-0.16)	<0.001	0.18 (0.14-0.27)	0.14 (0.10-0.19)	<0.001

Values are mean ± SD or median (IQR).

RHF = right heart failure; RVAD = right ventricular assist device; RVFW = right ventricular free-wall strain; RVGLS = right ventricular global longitudinal strain; RV-PA = right-ventricular pulmonary artery; sPAP = systolic pulmonary arterial pressure; TAPSE = trans-annular plane excursion.

### Predicting the need for an RVAD and RHF

The summary of the univariable logistic regression analysis testing the association between the RVPA indexes and the need for an RVAD or RHF is presented in Table 3. Reduced RVFW strain (OR: 1.37; 95% CI: 1.23-1.55; *P* < 0.001) and RVGLS (OR: 1.55; 95% CI: 1.34-1.83; *P* < 0.001) were associated with the need for an RVAD. TTE-derived RVPA coupling defined by RVFW/sPAP (OR: 0.03^−4^; 95% CI: 0.03^−6^ to 0.01^−2^; *P* < 0.001) and by RVGLS/sPAP (OR: 0.09^−6^; 95% CI: 0.01^−9^ to 0.06^−4^; *P* < 0.001) were both associated with the need for an RVAD. Among RHC hemodynamic variables, only pulmonary artery pulsatility index (PAPi) (OR: 0.67; 95% CI: 0.51-0.83; *P* < 0.001) and right atrial pressure (OR: 1.08; 95% CI: 1.01-1.14; *P* = 0.016) were associated with the need for an RVAD.

**Table 3:** Univariable Logistic Regression to Predict the Need for an RVAD or RHF

	RVAD		RHF	
	OR (95% CI)	*P* Value	OR (95% CI)	*P* Value
RVFW (%)	1.37 (1.23-1.55)	<0.001	1.26 (1.16-1.37)	<0.001
RVGLS (%)	1.55 (1.34-1.83)	<0.001	1.37 (1.23-1.54)	<0.001
RVFW/sPAP (%/mm Hg)	0.03^−4^ (0.03^−6^ to 0.01^−2^)	<0.001	0.04^−3^ (0.02^−4^ to 0.06)	<0.001
RVGLW/sPAP (%/mm Hg)	0.03^−6^ (0.05^−9^ to 0.06^−4^)	<0.001	0.02^−4^ (0.02^−6^ to 0.8^−3^)	<0.001
sPAP (mm Hg)	0.97 (0.94-1.01)	0.169	0.98 (0.95-1.01)	0.160
dPAP (mm Hg)	1.02 (0.97-1.08)	0.393	1.02 (0.97-1.06)	0.421
mPAP (mm Hg)	0.99 (0.95-1.04)	0.819	0.99 (0.96-1.03)	0.797
PCWP (mm Hg)	1.04 (0.98-1.10)	0.165	1.04 (0.98-1.09)	0.106
CI (L/min/m^2^)	0.90 (0.40-1.41)	0.739	1.15 (0.77-1.74)	0.435
PVR (WU)	0.89 (0.70-1.07)	0.307	1.04 (0.91-1.16)	0.544
RVESP (mm Hg)	0.99 (0.96-1.03)	0.577	0.99 (0.96-1.02)	0.414
RVEDP (mm Hg)	1.03 (0.96-1.10)	0.322	1.02 (0.96-1.08)	0.451
PAPi	0.67 (0.51-0.83)	<0.001	0.81 (0.69-0.91)	<0.001
RAP (mm Hg)	1.08 (1.01-1.14)	0.016	1.07 (1.02-1.30)	0.012

CI = cardiac index; dPAP = diastolic pulmonary arterial pressure; mPAP = mean pulmonary arterial pressure; PAPi = pulmonary artery pulsatility index; PCWP = pulmonary capillary wedge pressure; PVR = pulmonary vascular resistance; RAP = right atrial pressure; RHF = right heart failure; RVAD = right ventricular assist device; RVEDP = right ventricular end-diastolic pressure; RVESP = right ventricular end-systolic pressure; RVFW = right ventricular free-wall strain; RVGLS = right ventricular global longitudinal strain; RVGLW = right ventricular global longitudinal strain; sPAP = systolic pulmonary arterial pressure; TEE = trans-esophageal echocardiography; TTE = transthoracic echocardiography.

Concerning the associations with RHF, decreased RVFW strain (OR: 1.26; 95% CI: 1.16-1.37; *P* < 0.001) and RVGLS strain (OR: 1.23; 95% CI: 1.23-1.54; *P* < 0.001) were associated with the development of RHF. Strain-based RVPA coupling indexes RVFW/SPAP (OR: 0.04^−3^; 95% CI: 0.01^−4^ to 0.06; *P* < 0.001) and RVGLS/sPAP (OR: 0.02^−4^; 95% CI: 0.02^−6^ to 0.08^−2^; *P* < 0.001) were both associated with the development of RHF. From the RHC parameters, PAPi (OR: 0.81; 95% CI: 0.69-0.91; *P* < 0.001) and right atrial pressure (OR: 1.07; 95% CI: 1.02-1.30; *P* = 0.012) were associated with RHF.

**Figure 2:**
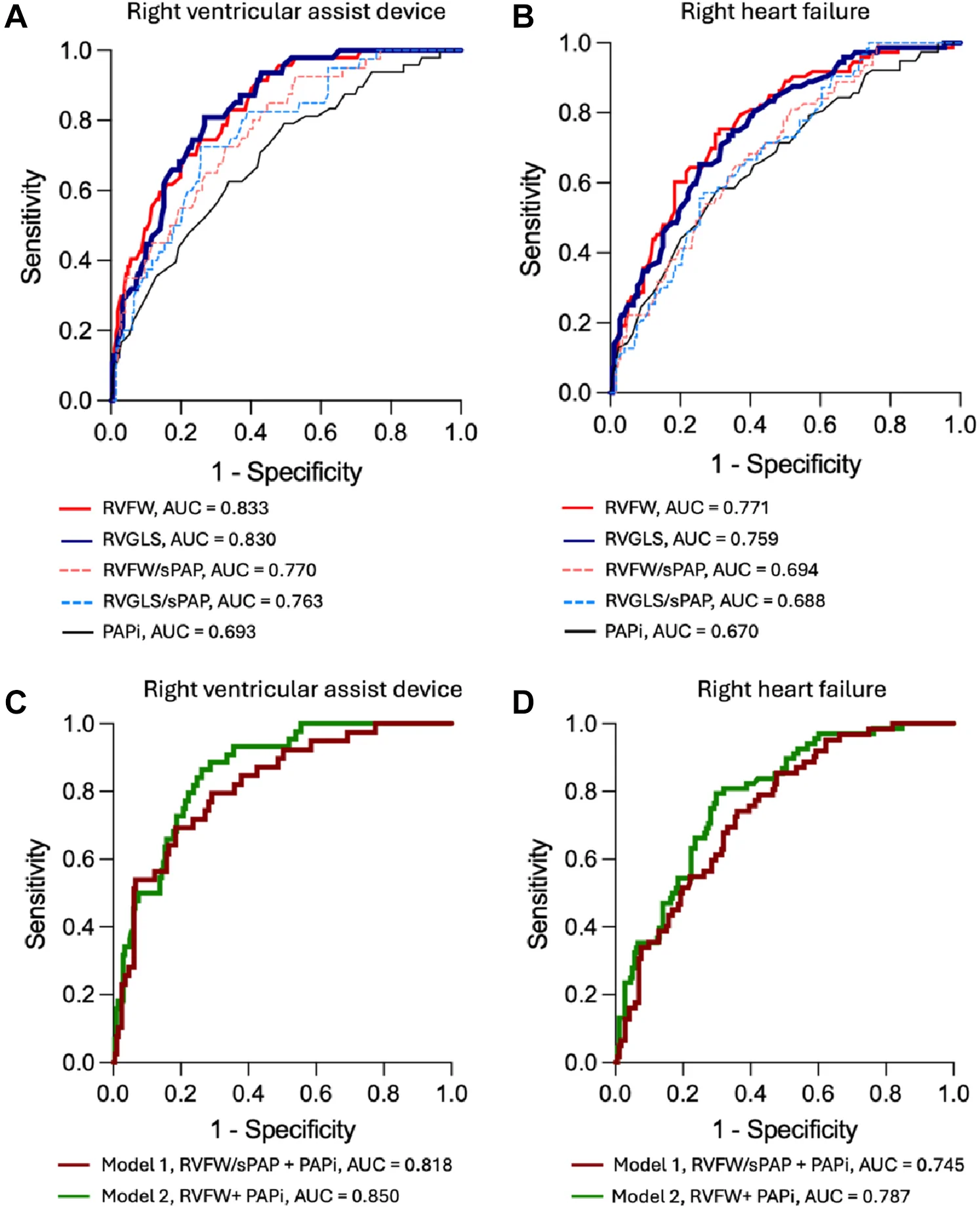
Echocardiographic and Catheterization-Derived Models Receiver operating curves demonstrating the predictive accuracy of risk single-variable (A and B) and multivariable (C and D) models for the need for a right ventricular assist device and right heart failure. AUC = area under the curve; PAPi = pulmonary artery pulsatility index; RVFW = right ventricular free-wall strain; RVGLS = right ventricular global longitudinal strain; sPAP = systolic pulmonary artery pressure.

The prognostic accuracy of variables significantly associated with the need for an RVAD or RHF is summarized in Table 4 with corresponding receiver operating curves analyses depicted in Figure 2. Using a backward multivariable regression approach with only nonlinear covariates predictive of the need for an RVAD or RHF, we developed 2 separate models combining echocardiographic strain and RHC indexes. Model 1 consisted of RVPA coupling using RVFW/sPAP and PAPi, and model 2 included RVFW strain and PAPi. We report these 2 models separately to compare one model using the RVPA coupling index and one with a variable assessing the RV systolic function.

**Table 4:** Prognostic Accuracy of Models in Validation Cohort to Predict the Need for an RVAD or RHF

	Sens	Spec	PPV	NPV	AUC	*P* Value
Need for RVAD						
RVFW	85.3	61.7	72.2	85.5	0.833	<0.001
RVGLS	73.2	80.8	61.4	84.6	0.832	<0.001
RVFW/sPAP	72.5	69.1	57.1	78.8	0.770	<0.001
RVGLS/sPAP	72.5	65.5	50.5	83.5	0.763	<0.001
PAPi	60.4	68.3	61.2	72.4	0.693	<0.001
Model 1: RVFW/sPAP + PAPi	79.5	77.5	40.8	83.5	0.818	<0.001
Model 2: RVFW + PAPi	88.6	71.3	70.2	86.7	0.850	<0.001
Right heart failure						
RVFW	73.9	70.1	64.5	76.1	0.771	<0.001
RVGLS	63.9	74.5	60.6	76.3	0.759	<0.001
RVFW/sPAP	65.1	65.3	55.1	73.4	0.694	<0.001
RVGLS/sPAP	55.5	74.5	56.7	74.5	0.688	<0.001
PAPi	57.0	70.3	62.9	65.2	0.670	<0.001
Model 1: RVFW/sPAP + PAPi	59.6	77.9	63.6	75.3	0.745	<0.001
Model 2: RVFW + PAPi	84.1	62.1	67.6	78.4	0.787	<0.001

AUC = area under the curve; NPV = negative predictive value; PAPi = pulmonary artery pulsatility index; PPV = positive predictive value; RHF = right heart failure; RVAD = right ventricular assist device; RVFW = right ventricular free-wall strain; RVGLS = right ventricular global longitudinal strain; Sens = sensitivity; sPAP = systolic pulmonary artery pressure; Spec = specificity.

For RVAD need, model 1 (RVFW/sPAP and PAPi) yielded an area under the curve (AUC) of 0.818 with a sensitivity of 79.5%, a specificity of 77.5%, a positive predictive value of 40.8%, and a negative predictive value of 83.50%. Model 2, composed of RVFW and PAPi, provided an AUC of 0.850 with a sensitivity of 88.6%, a specificity of 71.3%, a positive predictive value of 70.2%, and a negative predictive value of 86.7%. With regard to the RHF, model 1 resulted in an AUC of 0.745 with a sensitivity of 59.6%, a specificity of 77.9%, a positive predictive value of 63.6%, and a negative predictive value of 75.3%. Model 2 utilizing RVFW and PAPi resulted in an AUC of 0.787, a sensitivity of 84.1%, a specificity of 62.1%, a positive predictive value of 67.6%, and a negative predictive value of 78.4%.

### Survival analysis

**Figure 3:**
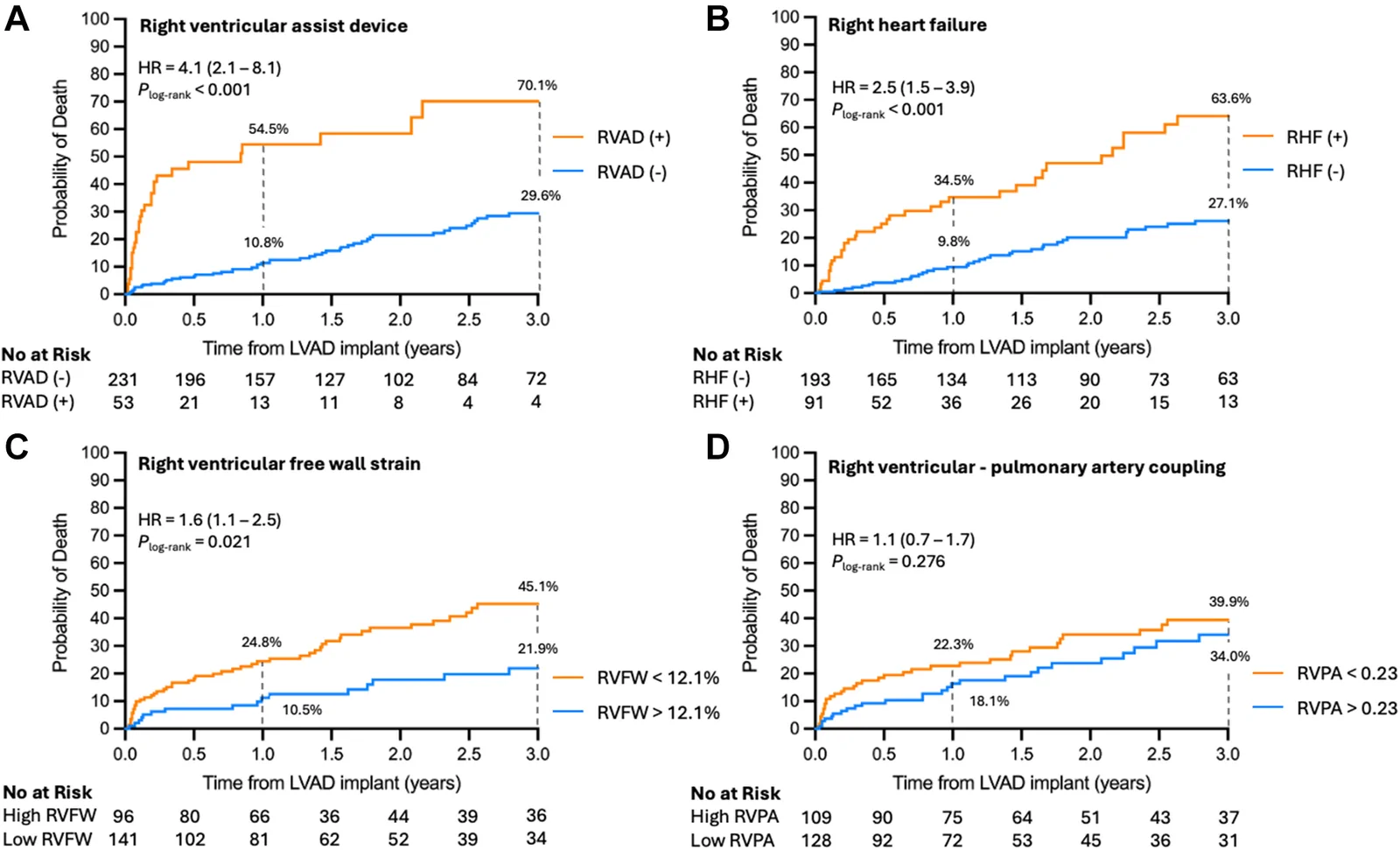
Survival Analysis Kaplan-Meyer survival curves demonstrating the significant effect on worse mortality with the need for an RVAD (A) and the development of RHF (B). Patients with preoperatively decreased RVFW strain values <12.1% had also worse mortality (C), but no significant effect was observed when considering the RVPA (D). LVAD = left ventricular assist device; RHF = right heart failure; RVAD = right ventricular assist device; RVFW = right ventricular free-wall strain; RVPA = RV pulmonary artery.

Survival curves for the entire patient population are depicted in Figure 3. Median follow-up time was 519 days (IQR: 185-1,129 days). Both the need for an RVAD and RHF were strongly associated with worse mortality. The need for an RVAD was associated with increased mortality at 1-year (54.5% vs 10.8%) and 3-year (63.6% vs 21.0%) follow-up, with an overall risk ratio of 4.1 (95% CI: 2.1-8.1; *P* < 0.001). Post-LVAD-implantation RHF was also associated with poor survival (34.5% vs 9.8%) at 1 year and 3 years (63.6% vs 21.0%) with an overall risk ratio of 2.5 (95% CI: 1.5-3.9; *P* < 0.001). RVFW values worse than −12.1% (Youden index cutoff value) were also associated with overall worse survival at 1 year (24.8% vs 10.5%) and 3 years (45.1% vs 21.9%) and an overall risk ratio of 1.6 (95% CI: 1.1-2.5; *P* < 0.001). RVPA coupling defined by RVFW/sPAP was not associated with worse mortality. In univariable Cox proportional hazards regression, RVAD was associated with worse mortality (HR: 2.16; 95% CI: 1.40-3.32; *P* < 0.001), and the presence of RHF was nearly associated with worse mortality (HR: 1.34; 95% CI: 0.96-1.87; *P* = 0.084). Other hemodynamic factors and imaging indexes were not significantly associated with increased mortality in Cox proportional hazards survival models.

## Discussion

The study demonstrated that reduced RV strain indexes and worse RVPA coupling, derived from strain and sPAP measured by RHC, can characterize preoperative RV dysfunction before MCS implantation and predict the need for an RVAD and acute RHF. In addition, 2 models combining RVFW/sPAP and RVFW strain with PAPi showed improved predictive accuracy for the need for an RVAD and RHF. Interestingly, isolated strain-based measurements, particularly the RVFW strain, were stronger predictors of RHF and the need for an RVAD than RVPA coupling metrics. Overall, in this study focusing solely on imaging and RHC biomarkers, we were able to develop models with encouraging c-statistic using only 2 input variables characterizing the RV systolic function and RV loading conditions across multiple LVAD generations. These single-center cohort-based models will certainly require external validation, but given the current wide clinical availability of myocardial strain imaging, a prospective multicenter external validation study should be implemented.

The majority of recently proposed risk-prediction models, which have been subjected to external validation including EUROMACS,^15^ CRITT,^16^ Michigan,^17^ Utah,^18^ Penn,^19^ and the Pittsburgh decision tree,^20^ use as an imaging input qualitative or semiquantitative markers of the RV dysfunction or do not include them at all. However, there is a considerable discordance between the categorical or semiquantitative grading variables and continuous state-of-the-art measurements of RV function, such as strain deformation indexes.^21^ RV strain parameters, including the RVFW, are more reflective of loading conditions^22^ and correlate better with clinical outcomes in patients with pulmonary hypertension.^23^^,^^24^ The primary hesitation to routinely apply RV strain parameters in a clinical setting or standard pre-LVAD assessment is the need for good-quality images and an optimal acoustic window for accurate speckle-tracking analysis of the RV free wall. Indeed, a recent meta-analysis demonstrated only a marginally significant effect of RVFW strain in predicting RHF post LVAD implantation, mainly due to the high variability of strain values.^25^ Our results show that RVFW strain provides important clinical value in predicting RHF and the need for an RVAD, in addition to showing value in predicting long-term survival. The cutoff value derived by RVFW of −12.1% was remarkably close to the value recently reported by Aymami (−12.7%) for predicting the RHF.^21^ Grant et al calculated that an RVGLS value <−9.6% was incremental to the Michigan risk score as a predictor of RV failure by increasing the model c-statistic from 0.66 to 0.77.^26^ Not surprisingly, TAPSE and TAPSE-based RVPA coupling did not show predictive potential, as supported by a recent meta-analysis that found TAPSE to be nonsignificant and highly heterogeneous in prognostic value.^25^ Lastly, there is a lack of large-scale imaging studies focusing on RV strain-based parameters in this patient population^27^ despite current clinical availability of strain-based postprocessing technologies.

Characterization of RVPA coupling in patients with durable MCS using traditional pressure-volume analysis provides invaluable insights into the RV's adaptive potential. Two recent studies elegantly demonstrated the limited ability of the RV to increase contractility in the setting of elevated afterload in patients with an LVAD.^7^^,^^8^ Consequently, a better understanding of RV function under different loading conditions is critical before considering MCS. Imaging-based estimates of RVPA coupling have shown potential for phenotyping patients with pulmonary hypertension^28^, ^29^, ^30^ and have demonstrated significant clinical prognostic value.^11^^,^^31^ In our study, we found that TTE-derived preoperative RVPA coupling indexes, RVFW/sPAP and RVGLS/sPAP, are decreased in patients who developed RHF or required an RVAD, suggesting a pre-existing limitation of the RV to adapt to changing loading conditions following LVAD implantation. However, we found that both coupling indexes performed worse than their respective RVFW and RVGLS strain values in univariable and multivariable models. sPAP derived from RHC was not associated with worse outcomes in this or prior studies investigating RHF following MCS implantation.^32^^,^^33^ Pulmonary arterial elastance, defined as the ratio of sPAP to stroke volume, is considered a more comprehensive RV afterload measurement and has been shown to be a better predictor of RHF post MCS implantation.^33^ In summary, simplified RVPA coupling indexes using sPAP might not be ideal before MCS evaluation, and additional RV afterload factors, including pulmonary vascular stiffness and postcapillary hemodynamics, should be thoroughly evaluated before MCS implantation. Lastly, the predictive ability of RV strain and RVPA coupling indexes was more precise for the need for an unplanned RVAD procedure than for the RHF. This is not surprising, as postoperative care practices and the clinicians titrating inotropic support might vary over time.

Similar to the previous studies, the need for an RVAD or the development of RHF was associated with significantly worse mortality.^34^^,^^35^ The rate of RHF in our study was 28.5%, and the rate of RVAD placement was 18.5%. Recent results from the European registry showed that the RHF rate was 21.4%, with a slightly higher prevalence in women.^36^ In addition, preoperatively reduced RVFW strain was associated with a higher mortality. Following LVAD implantation, late-onset RHF is thought to be either a primary process of insidious progression of pre-existing RV dysfunction or progression of pulmonary vascular disease as a result of continuous precapillary and/or postcapillary vascular remodeling.^37^ Although altered loading conditions after LVAD implantation might lead to the recovery of RV function,^38^ pre-existing irreversible structural changes to the RV myocardium might limit the functional improvement in the setting of limited ability to increase contractility.^7^ Therefore, more thorough preoperative characterization of the pulmonary vascular biomechanics using impedance or wave-intensity analysis might be warranted to characterize additional RV afterload components and the risk of future RHF.^39^^,^^40^ Compared with the RVFW strain, the state of preimplant RVPA coupling was not associated with mortality. This is likely because of an acutely changed hemodynamic profile post LVAD implantation, which should lead to reduced steady-state pulmonary vascular resistance. Importantly, an increasing number of studies are focusing on optimizing RV function before LVAD implantation, and identifying reliable preoperative imaging variables predictive of RHF might help to guide pre-LVAD implantation RV conditioning.^41^

### Study Limitations

The limitations of this study are primarily related to its retrospective single-institution design. The resulting models warrant external validation but include variables routinely or easily obtainable from the preoperative assessment before LVAD implantation. In addition, this was a focused study that considered standard RHC hemodynamic variables and echocardiographic imaging parameters; therefore, we omitted comparisons with other RHF risk models and variables. For the same reason, we avoided a potentially premature comparison of c-statistic values with those of other known models. We included all patients within a 10-year period during which clinical practice and quality of image acquisition have changed. However, RV myocardial strain imaging in patients with a good acoustic window is considered to have great intervendor and interreader reproducibility.^14^ Lastly, the 10-year period covered different types of MCS devices and likely different intraoperative and postoperative strategies, for which we could not adjust our analyses.

## Conclusions

Preoperative echocardiographic RV strain imaging can aid in predicting the need for RVAD and RHF following LVAD implantation (Central Illustration). Developed risk models included only 2 variables, RVFW strain and PAPi, reflective of systolic RV performance and its ability to adapt to different loading conditions. Overall, a more comprehensive assessment of the RVPA axis and RV myocardial deformation before LVAD implantation is warranted, particularly when upfront RV protection strategies pre- and post-LVAD implantation have been shown to result in significantly greater survival rates.^42^^,^^43^

Perspectives **COMPETENCY IN MEDICAL KNOWLEDGE:** Extending the preoperative evaluation before LVAD implantation to right ventricular strain–based evaluation might help to identify patients at risk of developing right heart failure and the need for a simultaneous right ventricular assist device. Strain-based echocardiographic analysis is now widely clinically available and should be part of routine preoperative reporting.

**TRANSLATIONAL OUTLOOK:** The vast majority of predictive right heart failure risk score models do not use quantitative measurements of RV function as input variables. This study suggests that RV strain-based parameters should be prospectively validated as predictors of right heart failure and subsequently incorporated into larger multivariable or artificial intelligence models to enhance their predictive yield.

**Central Illustration:**
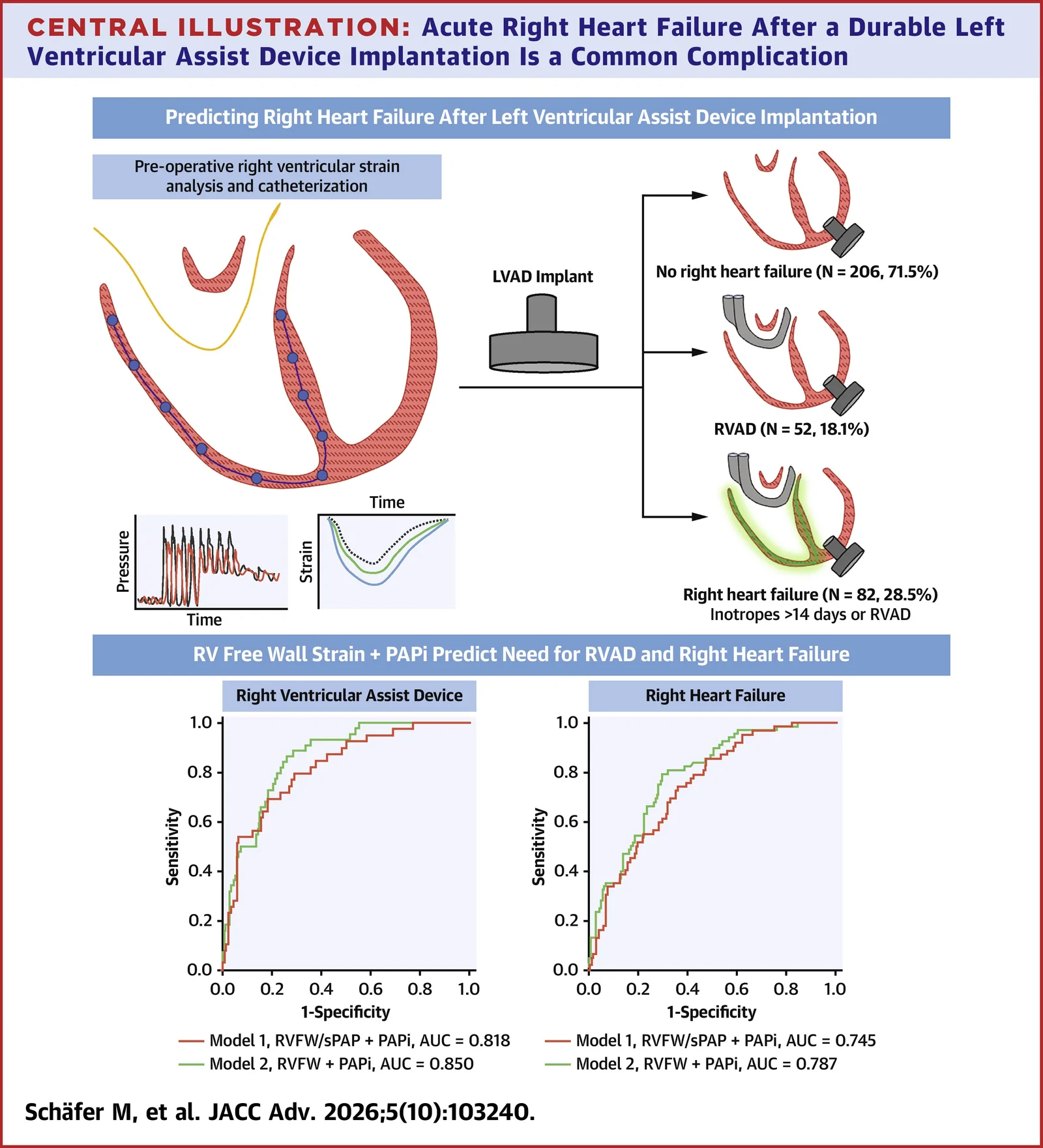
Acute Right Heart Failure After a Durable Left Ventricular Assist Device Implantation Is a Common Complication Predicting the right heart failure is of critical clinical importance because planned bi-ventricular support can yield better clinical outcomes. In this study, we focused on applying right ventricular strain analysis and catheterization hemodynamic parameters to predict right heart failure in a large cohort of patients. AUC = area under the curve; LVAD = left ventricular assist device; PAPi = pulmonary artery pulsatility index; RV = right ventricle; RVAD = right ventricular assist device; RVFW = right ventricular free-wall strain; sPAP = systolic pulmonary arterial pressure.

## Funding support and author disclosures

The authors have reported that they have no relationships relevant to the contents of this paper to disclose.
